# Postoperative atrial fibrillation in paraesophageal hernia repair: can it be prevented?

**DOI:** 10.1186/s13741-021-00191-7

**Published:** 2021-07-20

**Authors:** Jennifer Mardini, Melanio Bruceta, William Parrella-O’Donnell, Kunal Karamchandani

**Affiliations:** 1 Penn State College of Medicine, Hershey, PA 17033 USA; 2 Department of Anesthesiology & Perioperative Medicine, Penn State College of Medicine, 500 University Ave, Hershey, PA 17033 USA

**Keywords:** Atrial fibrillation, Hypertension, Calcium channel blocker, Paraesophageal hiatal hernia, Acute ischemic stroke

## Abstract

Acute ischemic thromboembolic stroke is one of the most feared complications of atrial fibrillation (AF), and the risk increases with higher CHA2DS2-VASc scores. Postoperative atrial fibrillation (POAF) is common after noncardiac surgery, particularly after thoracic surgery, and can result in significant morbidity and mortality. We report the case of an 85-year-old female with a history of untreated hypertension (HTN) and no prior history of AF, who presented 5 days after an elective repair of a paraesophageal hernia with recurrence of a large type III paraesophageal hiatal hernia, AF, and subsequent acute thromboembolic ischemic stroke. Patient’s AF resolved shortly after treatment with calcium channel blocker. The risk of stroke is high in patients who develop AF and a period of 48 h after onset of AF is usually considered safe as the risk of stroke is low in this time period. However, this may not be the case during the perioperative period and preventive measures such as preoperative calcium channel blocker could be considered. Our case highlights that acute ischemic thromboembolic stroke might develop earlier tha 48 h after onset of POAF in patients undergoing paraesophageal hernia repair. Initiation of a calcium channel blocker should be considered during preoperative evaluation for patients undergoing paraesophageal hernia repairs, especially in those with untreated HTN.

## Introduction

Postoperative atrial fibrillation (POAF) is a common condition ensuing noncardiac surgery, with a reported incidence rate between 0.4 and 3.0% (Polanczyk et al. 1998). Thoracic, pulmonary, vascular, and abdominal surgeries are associated with the highest risk (Butt et al. 2018). In particular, it has been reported that hiatal hernias appear to be associated with increased frequency of atrial fibrillation (AF) in both men and women of all ages due to mechanical compression of the atria (Roy et al. 2013). Other risk factors have been associated with POAF after noncardiac surgery, which have led to the identification of patients benefiting from pharmacologic or alternative prophylaxis (Vaporciyan et al. 2004). In particular, hypertension is associated with a 1.8-fold increase in the risk of developing POAF as it predisposes to cardiac structural changes that influence the development of AF (Ogunsua et al. 2015). L-type calcium channel blockers have become a common treatment modality in some institutions for prophylaxis of AF in high-risk patients undergoing major thoracic surgery (Amar et al. 2000). Currently, there are no set recommendations for medication prophylaxis in patients at high risk for POAF (Frendl et al. 2014).

We report the case of an 85-year-old female with history of untreated hypertension (HTN) who presented on post-op day five from paraesophageal hernia with recurrent large type III paraesophageal hiatal hernia, POAF, and subsequently developed acute thromboembolic ischemic stroke. The patient’s AF resolved shortly after administration of diltiazem, a non-dihydropyridine calcium channel blocker. We would like to highlight the potential use of POAF prophylaxis in high-risk patients undergoing paraesophageal hernia repair surgery, and propose a possible treatment regimen with non-dihydropyridine calcium channel blocker, particularly in those with untreated or uncontrolled HTN.

## Case synopsis

An 85-year-old female with a past medical history significant for renal cell carcinoma, gastroesophageal reflux disease, and untreated hypertension presented to the intensive care unit on postoperative day five from a laparoscopic large type III paraesophageal hiatal hernia repair with gastropexy, with right-sided weakness, mild dysarthria, and right hemineglect (National Institute of Health Stroke Scale (NIHSS) = 11). Upon arrival to the intensive care unit, she had a heart rate of 120–130 beats per minute, respiratory rate of 20–30 breaths per minute, oxygen saturation > 92% on room air, and blood pressure ranging from 160 to 180 mmHg systolic and 90–100 mmHg diastolic. The patient had been discharged 3 days after her surgery with cardiac telemetry showing normal sinus rhythm.

A computed tomography of the head without contrast was performed and ruled out acute hemorrhage; however, tissue plasminogen activator was contraindicated in the setting of recent surgery. A magnetic resonance imaging of the brain exhibited scattered acute infarcts in bilateral frontal lobes, and subsequent head and neck magnetic resonance angiography revealed bilateral anterior cerebral artery occlusion.

An electrocardiogram was performed and exhibited AF with rapid ventricular response of 120 bpm and a blood pressure of 165/90. The patient was immediately treated with diltiazem 10 mg intravenous followed by a diltiazem infusion with a resolution of the AF within 2 h which lead to discontinuation of the drip. Subsequent rhythm strips for the following 12 h showed normal sinus rhythm. The following day, the patient had another episode of AF with a rapid ventricular response, at this time, it was decided to use amiodarone to prevent any potential hemodynamic instability in the setting of a recent acute ischemic stroke. The patient was given amiodarone 150 mg intravenous and she was then placed on 3 days of amiodarone 200 mg twice daily through her nasogastric tube. A heparin drip with a partial thromboplastin time goal of 50–70 was also started for anticoagulation. A transthoracic echocardiogram with bubble study was performed and revealed moderate left atrial dilation. However, no clots were seen in the left atrial appendage.

The patient was taken to the operating room for flexible esophagogastroduodenoscopy, gastric decompression, and placement of nasogastric tube when further imaging indicated rightward mediastinum shift due to recurrence of large type III paraesophageal hiatal hernia. The large type III hernia contained the entirety of the patient’s stomach, proximal duodenum, and transverse colon. The patient returned to the operating room 11 days after initial repair for repeat paraesophageal hiatal hernia repair as well as chest tube and percutaneous endoscopic gastrostomy tube placement. Heparin drip was held 12 h pre-operatively. Postoperative chest radiograph indicated resolution of mediastinal shift with re-expansion of the left lung. After her initial 3 days loading dose of amiodarone, the patient was continued on a 200-mg daily dose through her perioperative period to prevent additional episodes. She was discharged on amiodarone 200 mg daily and apixaban 2.5 mg twice daily 17 days after her admission to a long-term care facility.

## Case discussion

AF is a common acute cardiac disorder associated with significant morbidity and mortality, with acute ischemic thromboembolic stroke being one of the most serious complications (Staerk et al. 2017). Risk prediction models, such as the CHA_2_DS_2_-VASc clinical stroke risk score, have been developed to aid with selecting those at high risk for embolic stroke with recommended anticoagulation for those with scores ≥ 2 in men and ≥ 3 in women (Olesen et al. 2011; Hindricks et al. 2021). Although the CHA2DS2-VASc score has not been validated in the postoperative population, anticoagulation is usually indicated for higher score patients with multiple episodes of AF or a single episode lasting > 24–48 h (Hart et al. 2007).

It is possible that both the recurrence of the hiatal hernia and her history of uncontrolled hypertension may have been the principal causes of AF in this patient and thus subsequent embolic stroke, although such conclusions cannot be proven. POAF is known to occur in association with numerous acute conditions such as hypo- and hypervolemia, electrolytes imbalances, anemia, infection, myocardial infarction, hypoxia, hypoglycemia, and intraoperative hypotension (Vaporciyan et al. 2004). This patient lacked any of the aforementioned conditions. Our patient did have left atrium enlargement which is known to increase the risk of thrombus formation in the left atrial appendage, as well as gastroesophageal reflux disease with inflammation and vagal stimulation playing a possible role (Toufan et al. 2017; Roman et al. 2014). This finding, tied in with her high-risk status (CHA_2_DS_2_-VASc > 2 and thoracic surgery), would allow this patient to benefit from prophylactic treatment with a rate-control agent such as diltiazem prior to surgery and avoid the risk of stroke sequelae.

The effect of prophylactic L-type calcium channel blockers has been studied after major thoracic operations and demonstrated nearly 50% reduction in the incidence of POAF by reducing the heart rate early after operation as well as the ventricular response rates during AF (Amar et al. 2000). While Amar et al. did not show any significant difference in mortality between the placebo and the diltiazem group, it has been shown that the incidence of POAF increases mortality, hospital costs, and readmission rates (LaPar et al. 2014). In assessing the risk-benefit profile of diltiazem in the short-term, it is reasonable to administer it to those intermediate to high-risk patients preoperatively in order to prevent POAF (Frendl et al. 2014). Other pharmacological therapies have been studied for the prevention of POAF in noncardiac surgery with most favorable results with the use of beta blockers, amiodarone, statins, and calcium channel blocker (Oesterle et al. 2018). However, calcium channel blocker has been shown to have the most positive results with the least side effect profile in thoracic surgery.

It is unfortunate that the patient was not initially maintained on a rate control agent following conversion to sinus rhythm after presenting in new onset atrial fibrillation. However, this decision was outside the scope of our direct care. Nonetheless, given her initial presentation of acute ischemic stroke in the absence of revascularization, it can be easily presumed that this clinical decision was geared towards the prevention of drastic hemodynamic changes and optimization of collateral cerebral blood flow.

The incidence of AF has been reported to be significantly high (7.1%) even in low-risk patients undergoing hiatal hernia repair (Roy et al. 2013). Although paraesophageal hiatal hernia repairs were not specifically included in this study, one can deduce that mass effect of abdominal contents into the chest cavity can directly impinge on the left atrium. It is difficult to predict if prophylactic diltiazem would have prevented the atrial fibrillation in this case given the large size of the recurrent hernia. However, it is an illustrative case of the potential catastrophic outcomes that can be associated with new onset atrial fibrillation and underlines the importance of appropriate prophylactic measures.

Previous evidence suggests that transient POAF after non-cardiac surgery may carry a long-term stroke risk similar to any other AF diagnosis (Gialdini et al. 2014). In this case, the patient developed a stroke so quickly that she was not able to be adequately anticoagulated and so the best way to tackle this type of patients would be with prevention measures. It is possible that those patients at high risk of hiatal hernia recurrence and high CHA_2_DS_2_-VASc score could benefit from calcium channel blocker prophylaxis; however, further studies should be performed.

## Conclusion

Acute ischemic thromboembolic stroke might develop earlier that 48 h in postoperative paraesophageal hernia repair patients with POAF. Although HTN is not the sole independent risk factor, preventive measures such as calcium channel blocker should be considered during preoperative evaluation for patients undergoing paraesophageal hernia repairs, especially in patients with untreated HTN.

## Data Availability

Not applicable.

## Abbreviations

AF: Atrial fibrillation
POAF: Postoperative atrial fibrillation
HTN: Hypertension
